# Prevention, Treatment and Diagnosis of Tuberculosis, 2nd Edition

**DOI:** 10.3390/microorganisms14010163

**Published:** 2026-01-12

**Authors:** Rogelio Hernández Pando

**Affiliations:** Instituto Nacional de la Nutrición Salvador Zubiran, Tlalpan 10480, Mexico; rogelio.hernandezp@incmnsz.mx

Tuberculosis (TB) remains a global health challenge, partly due to difficulties in diagnosis and the prolonged duration and toxicity of standard antibiotic regimens. After being replaced by coronavirus disease (COVID-19) for three years, TB has now regained its position as the world’s leading infectious agent-related cause of death. The disease holds a hugely significant place in human history due to the magnitude of morbidity and mortality it has brought since the beginning of civilization [1]. Every year, more than 10 million cases of active TB and 1.2 million deaths are reported [2]. Treatment requires six months of a combination of antibiotics, which is a significant obstacle to eradication [3]. Significant progress in drug development during the last decade greatly contributed to TB therapy, but other kinds of strategies should be considered to further improve the control of the disease. Particular attention needs to be paid to the increasing emergence of drug-resistant strains of M. tuberculosis (MDR), as well as the association of TB with HIV infection and with chronic degenerative diseases, such as type II diabetes. This book contains a collection of 10 papers that advance the field of TB prevention, treatment, and diagnosis.

The COVID-19 pandemic significantly disrupted TB control efforts, leading to delays in diagnosis, underreporting of deaths and positive cases, and increased loss to follow-up of active cases. Consequently, TB mortality increased, especially among vulnerable populations. This trend was clearly demonstrated in the study by Tavares et al. [4], which used the national TB mortality data from Brazil and employed a Bayesian approach; a significant increase in TB mortality (estimated at 9.9%) during the COVID-19 pandemic was revealed, with notable disparities affecting females, elderly individuals, indigenous and black populations. These findings highlight the need for policymakers to address these health inequities in the design of strategies aiming to decrease the pandemic’s impact on TB mortality.

Type 2 diabetes mellitus (T2DM) is the most common form of DM, accounting for approximately 90% of cases worldwide. Diabetic patients are more susceptible to infections due to an impaired immune system and TB is a particularly important comorbidity since T2DM increases the risk of developing active TB and exacerbates disease severity. Moreover, six of the ten countries with the most T2DM cases are classified by the WHO as “high-burden” TB countries, accounting for approximately 80% of all TB cases worldwide. Cornejo-Báez et al. reviewed the mechanisms by which T2DM-induced metabolic and immune dysregulation increases the survival of Mtb, as well as promotes the persistence and emergence of multidrug-resistant (MDR) strains, another global concern highlighted by the WHO [5]. Although the treatment of drug-sensitive TB generally has good recovery rates, the T2DM condition may complicate the clinical situation via participation in MDR induction, particularly those resistant to the most efficient primary drugs: isoniazid (INH) and rifampicin (RIF). Indeed, drug resistance is strongly associated with glycaemic control in T2DM. This review considers recently described mechanisms that contribute to MDR, such as efflux pump activation, altered expression of transcriptional regulators, enhanced DNA repair systems, and altered drug pharmacokinetics. Authors have also proposed innovative strategies such as efflux pump inhibitors and host-directed therapies to combat T2DM–TB comorbidity and its role in drug resistance. Closely related to this important subject is the work of Chimal Muñoz et al. [6], who analysed almost 400 Mtb genomes from bacteria obtained from individuals with or without T2DM and with or without DR. Of the 164 efflux pump genes analysed, 10 lacked any variant, while 154 presented between 3 and 19 variants. The variant S217P in mmpL13a (Rv1145) was the most common, being found in 98 (25%) isolates. The authors observed a significant association between 19 variants and DR, and between 20 variants and T2DM (*p* ≤ 0.005). Thus, it seems that there are certain TB variants that are more common in individuals with DR and T2DM, suggesting a possible influence of the host on TB evolution.

The prolonged treatment duration of TB is associated with adverse effects that negatively affect patient adherence, reducing the effectiveness of the therapy and contributing to the emergence of MDR strains. MDR TB is a significant challenge due to its high lethality and the complexity and cost of treatment. Thus, the development of novel synthetic drugs and natural products derived from medicinal plants represents a promising approach that could be effective against MDR-TB or help to reduce the duration of conventional chemotherapy. Natural products derived from plants have been used as alternative treatments for many diseases. Usually, herbal medicines are a combination of multiple components with similar properties that often show synergistic effects, such as ursolic acid (UA) and oleanolic acid (OA), two pentacyclic triterpenoid isomers that, when used alone or in combination in a macrophage cell line, exhibit a synergistic effect against drug-sensitive and MDR Mtb strains. Moreover, UA and OA exhibit hepatoprotective effects against the toxicity of INH and RIF; however, they are non-polar, strongly fat-soluble compounds with low oral bioavailability, which is a significant inconvenience for TB treatment. Therefore, improving the solubility and delivery of these triterpenes through nanotechnology is an interesting strategy. In a murine model of pulmonary MDR TB, Saini V et al. showed that solid lipid microparticles (SLMs) are efficient colloidal nanoparticles that serve as UA and OA transporters when administered directly to the lungs using a metered-dose inhaler [7]. This treatment, based on inhalation therapy with UA and OA, could be a viable adjuvant treatment for TB.

There are many types of nanoparticles currently being tested as carriers of antibiotics. Chitosan (poly-(1,4-β-Dglucopyranosamine) is derived from chitin, which is the second most abundant natural polymer after cellulose. Chitin is primarily found in the exoskeletons of crustaceans, the cell walls of fungi, and some insects. Its conversion to chitosan involves a deacetylation process where the acetyl groups are removed. Chitosan also has antimicrobial activity against a diverse array of microorganisms. Due to the large surface-area-to-volume ratio of chitosan nanoparticles, they have enhanced reactivity and interaction with target cells, improving their effectiveness in drug delivery and antimicrobial treatments. The work of Olivas-Flores et al. [8] shows the efficient in vitro antimicrobial activity of chitosan nanoparticles, dependent on their size, against clinical isolates of Mtb and other microorganisms (*E. coli*, *S. aureus*, *E. faecalis,* and *C. albicans*), as well as of aqueous extracts and lyophilized powders of allium (garlic) species [8]. After comprehensive cytotoxicity studies and in vivo evaluations of the final formulations, it can be concluded that the best treatment route would be the administration of these nanoparticles via inhaler devices.

The identification of drug targets for evaluating candidates or repurposing drugs is another important approach to synthesizing or identifying new drugs against Mtb. This approach was used by Tovar Nieto et al., who targeted the essential cell division protein FtsZ using an in silico pharmacological repositioning strategy [9]. They found four FDA-approved drugs that bind to the catalytic site of FtsZ, the antimicrobial activity and cytotoxicity of which were assessed on human monocyte-derived macrophages (MDMhs). Repurposed paroxetine and nebivolol exhibited antimycobacterial activity against both reference TB and MDR strains at a concentration of 25 μg/mL. Both drugs demonstrated a significant reduction (*p* < 0.05) in viable bacteria compared to the untreated group in an in vitro infection model.

Regarding TB diagnosis, the World Health Organization (WHO) has emphasized the need for non-sputum-based tests with diagnostic accuracy comparable to traditional sputum assays. This has thrust salivary diagnostic techniques into the limelight. In a meta-analysis, Darwish R et al. reviewed 16 studies analysing the role of saliva-based tests in active TB diagnosis [10]. Searching online databases, they found minimal diagnostic potential for interleukin determination. Only IL-17 levels were consistently reduced in active TB patients, while other ILs failed to distinguish active TB from healthy or non-TB respiratory disease cases. Most studies relied on the GeneXpert MTB/RIF assay on saliva, but none fulfilled the WHO guidelines for a non-sputum test.

In patients who are unable to produce sputum, like children, stool samples can be useful for pulmonary TB diagnosis. However, contamination of such specimens is a risk with mycobacterial culture. Tiang et al. [11] evaluated the novel decontamination method of power ultrasound (PU) in terms of mycobacterial isolation in stool samples (n = 650); these were collected and treated using NaOH-NALC or PU. A total of 32 (4.92%) samples treated with the NaOH-NALC method were culture-positive for Mtb (M.TB; n = 21, 3.23%) and nontuberculous mycobacteria (NTM; n = 11, 1.69%). Sixty-one (9.38%) treated with the PU method were culture-positive, including Mtb (n = 37, 5.69%) and NTM (n = 24, 3.69%), reaching statistical significance (*p* < 0.05). Compared with the NALC-NaOH method (19.07%), stool samples treated with PU (13.23%) showed a significantly lower contamination rate (*p* < 0.05). Thus, PU represents a novel decontamination technique that can significantly enhance the isolation of both NTM and Mtb in stool specimens for culture.

Usually, a primary Mtb infection is successfully controlled by the immune system. However, not all bacteria are eliminated; some remain in the tissues in a non-replicating or slowly replicating dormant state for the rest of the individual’s life. This condition is called latent TB (LTB). It is clinically asymptomatic and only manifests as an enduring immune response to Mtb antigens. One-fourth of the world’s population has this kind of quiescent infection, and in areas where TB endemicity is low, the majority of active cases surge as a result of dormant-bacilli reactivation. Accurate LTB diagnosis is not achievable through a single test and relies on the detection of cellular immune responses to Mtb antigens. In low-incidence settings, the public health focus is on identifying and treating LTB to prevent potential Mtb reactivation. Thus, early diagnosis of LTBI is important for reducing morbidity and preventing transmission to vulnerable individuals. Exosomes—small extracellular vesicles originating from diverse cell types—are potential diagnostic and therapeutic biomarkers. They contain diverse characteristic proteins involved in membrane fusion, signalling, and protein trafficking, as well as microRNAs (miRNAs) that are important regulators of the immune response to Mtb infection. Cui et al. conducted miRNA sequencing to determine the small RNA profiles of plasma exosomes from LTB individuals and healthy controls [12]. They identified 12 differentially expressed miRNAs; six (hsa-miR-7850-5p, hsa-miR-1306-5p, hsa-miR-363-5p, hsamiR-374a-5p, hsa-miR-4654, has-miR-6529-5p, and hsa-miR-140-5p) were more expressed in individuals with LTB whose targets were functionally related to ferroptosis and fatty acid metabolism, suggesting a critical role in regulating the intracellular survival of Mtb. Thus, the characterization of miRNAs in exosomes can help to identify potential molecular targets for the pathogenesis and diagnosis of LTB.

According to the WHO, the systematic testing and treatment of LTB should be carried out in the following groups: people living with HIV; adults and children who are contacts of pulmonary TB patients; individuals starting anti-tumour necrosis factor treatment (TNF); patients on dialysis, those preparing for organ transplantation, or those with silicosis; prisoners; healthcare professionals; homeless people; illicit drug addicts; and immigrants from countries with a high TB burden. For diagnosis, either interferon-gamma release assays (IGRAs) or the Mantoux tuberculin skin test (TST) should be used, and individuals positive for these conditions should be treated. One of the main problems is treatment adherence due to its prolonged duration. The Unified Health System (SUS) currently offers three therapeutic regimens that significantly reduce the antibiotic doses, permitting better treatment adherence with fewer adverse effects, but in migrants, it is difficult to monitor TB treatment during their journey to the destination. Mathias Alves et al. studied treatment adherence among international migrants and refugees in Manaus, Amazonas, Brazil, comparing three strategies: self-administration (SA), directly observed conventional therapy (DOT), and a video telemonitoring system (VDOT) [13]. The VDOT and SA groups exhibited the lowest rate of treatment dropout or interruption at 16.1%, followed by the DOT group at 23.1%. Thus, the most effective strategy for ensuring adherence among migrants and refugees was VDOT. Different treatment strategies should be adapted to the individuals’ needs and risk factors.

In conclusion, this collection of studies provides new information about the prevention, treatment, and diagnosis of TB. The COVID pandemic has contributed to an increase in TB mortality. It seems that type II diabetes is not only associated with a higher prevalence of more severe TB, but it also participates in the generation of MDR TB. Natural products, nanotechnology, and aerosol treatments are interesting new strategies to improve the treatment of drug-sensitive and -resistant TB. In addition to sputum, other sample types—such as stool—can be useful for diagnosis, particularly in children. However, these samples require special decontamination methods such as power ultrasound. Latent TB is a common condition that is difficult to diagnose and treat; some miRNAs located in exosomes can be used as markers of diagnosis. For efficient treatment adherence, the VDOT system can be of value, particularly in the migrant population.
